# Indoor and outdoor malaria vector surveillance in western Kenya: implications for better understanding of residual transmission

**DOI:** 10.1186/s12936-017-2098-z

**Published:** 2017-11-06

**Authors:** Teshome Degefa, Delenasaw Yewhalaw, Guofa Zhou, Ming-chieh Lee, Harrysone Atieli, Andrew K. Githeko, Guiyun Yan

**Affiliations:** 1 Department of Medical Laboratory Sciences, College of Health Sciences, Jimma University, Jimma, Ethiopia; 2 Centre for Global Health Research, Kenya Medical Research Institute, Kisumu, Kenya; 3 Tropical and Infectious Diseases Research Center (TIDRC), Jimma University, Jimma, Ethiopia; 4 Program in Public Health, College of Health Sciences, University of California at Irvine, Irvine, CA 92697 USA; 5 School of Public Health, Maseno University, Kisumu, Kenya

**Keywords:** Malaria vectors, Surveillance, Behavior, Residual transmission, Kenya

## Abstract

**Background:**

The widespread use of indoor-based malaria vector control interventions has been shown to alter the behaviour of vectors in Africa. There is an increasing concern that such changes could sustain residual transmission. This study was conducted to assess vector species composition, feeding behaviour and their contribution to indoor and outdoor malaria transmission in western Kenya.

**Methods:**

*Anopheles* mosquito collections were carried out from September 2015 to April 2016 in Ahero and Iguhu sites, western Kenya using CDC light traps (indoor and outdoor), pyrethrum spray catches (PSCs) (indoor) and pit shelters (outdoor). Species within *Anopheles gambiae* s.l. and *Anopheles funestus* s.l. were identified using polymerase chain reaction (PCR). Enzyme-linked immunosorbent assay (ELISA) was used to determine mosquito blood meal sources and sporozoite infections.

**Results:**

A total of 10,864 female *Anopheles* mosquitoes comprising *An. gambiae* s.l. (71.4%), *An. funestus* s.l. (12.3%), *Anopheles coustani* (9.2%) and *Anopheles pharoensis* (7.1%) were collected. The majority (61.8%) of the anopheline mosquitoes were collected outdoors. PCR result (n = 581) revealed that 98.9% *An. arabiensis* and 1.1% *An. gambiae* s.s. constituted *An. gambiae* s.l. in Ahero while this was 87% *An. gambiae* s.s. and 13% *An. arabiensis* in Iguhu. Of the 108 *An. funestus* s.l. analysed by PCR, 98.1% belonged to *An. funestus* s.s. and 1.9% to *Anopheles leesoni*. The human blood index (HBI) and bovine blood index (BBI) of *An. arabiensis* was 2.5 and 73.1%, respectively. *Anopheles gambiae* s.s. had HBI and BBI of 50 and 28%, respectively. The HBI and BBI of *An. funestus* was 60 and 22.3%, respectively. Forage ratio estimate revealed that *An. arabiensis* preferred to feed on cattle, *An. gambiae* s.s. showed preference for both human and cattle, while *An. funestus* preferred human over other hosts. In Ahero, the sporozoite rates for *An. arabiensis* and *An. funestus* were 0.16 and 1.8%, respectively, whereas in Iguhu, the sporozoite rates for *An. gambiae* s.s. and *An. funestus* were 2.3 and 2.4%, respectively. In Ahero, the estimated indoor and outdoor entomological inoculation rate (EIR) was 108.6 infective bites/person/year (79.0 from *An. funestus* and 29.6 from *An. arabiensis*) and 43.5 infective bites/person/year (27.9 from *An. arabiensis* and 15.6 from *An. funestus*), respectively. In Iguhu, the estimated indoor and outdoor EIR was 24.5 infective bites/person/year (18.8 from *An. gambiae* s.s. and 5.7 from *An. funestus*) and 5.5 infective bites/person/year (all from *An. gambiae* s.s.), respectively.

**Conclusion:**

*Anopheles gambiae* s.s. showed an increasing tendency to feed on cattle. *Anopheles arabiensis* was highly zoophagic, whereas *An. funestus* showed anthropophagic behaviour. While the majority of malaria transmission occurred indoor, the magnitude of outdoor transmission was considerably high. Additional control tools that complement the existing interventions are required to control residual transmission.

## Background

Malaria is a serious vector-borne disease affecting hundreds of millions of people in Africa. In the past decade, a substantial reduction in malaria incidence has been observed in Africa, including Kenya, due to the scale-up of interventions. Vector control is one of the key elements in achieving the remarkable decline of malaria, with the scale-up of insecticide-treated nets (ITNs) and expansion of indoor residual spray (IRS) contributing significantly [1–4]. The proportion of households owning at least one ITN in sub-Saharan Africa is estimated to have risen from 3% in 2000 to 67% in 2015 [1]. In western Kenya, the ITN ownership rose from 12.8% in 2004 to over 80% in 2015 [5–7].

Despite the progress made in scaling-up of the interventions, malaria transmission continues to occur. Several factors are responsible for this transmission, including the spread of insecticide resistance [5, 8], shift in vector species composition [9–12] and increasing vector behavioural change towards more zoophagic, exophagic and/or exophilic tendencies following the widespread use of ITNs and IRS [13, 14].

Recent reports from East Africa showed strong evidence for shifts in *Anopheles gambiae* sensu lato (s.l.) sibling species composition from predominantly endophagic *An. gambiae* sensu stricto (s.s.) to predominantly exophagic *Anopheles arabiensis* following the scale-up of ITNs [9, 11–13, 15]. In the lowlands of western Kenya, the proportion of *An. gambiae* s.s. declined from about 85% in 1998 to 1% in 2009 following massive distribution of ITNs, whereas *An. arabiensis* population showed proportionate increment [9]. While malaria transmission by *An. gambiae* s.s. declined significantly, residual transmission continued to occur by *An. arabiensis*. Similarly, the proportion of *An. arabiensis* in the highlands of western Kenya has been increasing gradually [5].

Vector behavioural modifications including changes in host-preference, biting locations (indoor or outdoor) and resting behaviours have been reported following the long-term use of ITNs. For instance, ITN use was associated with shift in host preference of *An. gambiae* s.s. from human to cattle in Burkina Faso [16]. The long-term use of ITN increased the outdoor feeding proportion of *An. gambiae* s.s. in Bioko Island [17, 18] and *Anopheles funestus* in Tanzania [13]. However, these changes are not universal. A recent study in Asembo district of western Kenya showed that the majority of biting by *An. arabiensis*, *An. gambiae* s.s. and *An. funestus* occurred indoors despite high ITN coverage in the area [19].

Malaria is mesoendemic and holoendemic in the highland and lowland areas of western Kenya, respectively [20]. The transmission is maintained by *An. gambiae* s.s., *An. funestus* and *An. arabiensis*. *Anopheles gambiae* s.s and *An. funestus* are considered as highly endophagic and anthropophagic, while *An. arabiensis* is considered as zoophagic and endophilic. However, most of the studies on their feeding and resting behaviour were conducted before the scale-up of vector control interventions [21–23]. It is possible that the anthropophagic and endophilic individuals could shift to zoophagic and exophilic tendencies or be reduced to leave zoophagic and exophilic sibling species following the scale-up of ITNs as has been observed elsewhere.

In view of the increasing concern about residual malaria transmission in Africa, there is a pressing need to enhance our understanding about vector behaviours to evaluate the likely success of the current vector control tools. The main aim of this study was to assess vector species composition, feeding behaviour and their contribution to indoor and outdoor malaria transmission in western Kenya.

## Methods

### Study sites

**Fig. 1:**
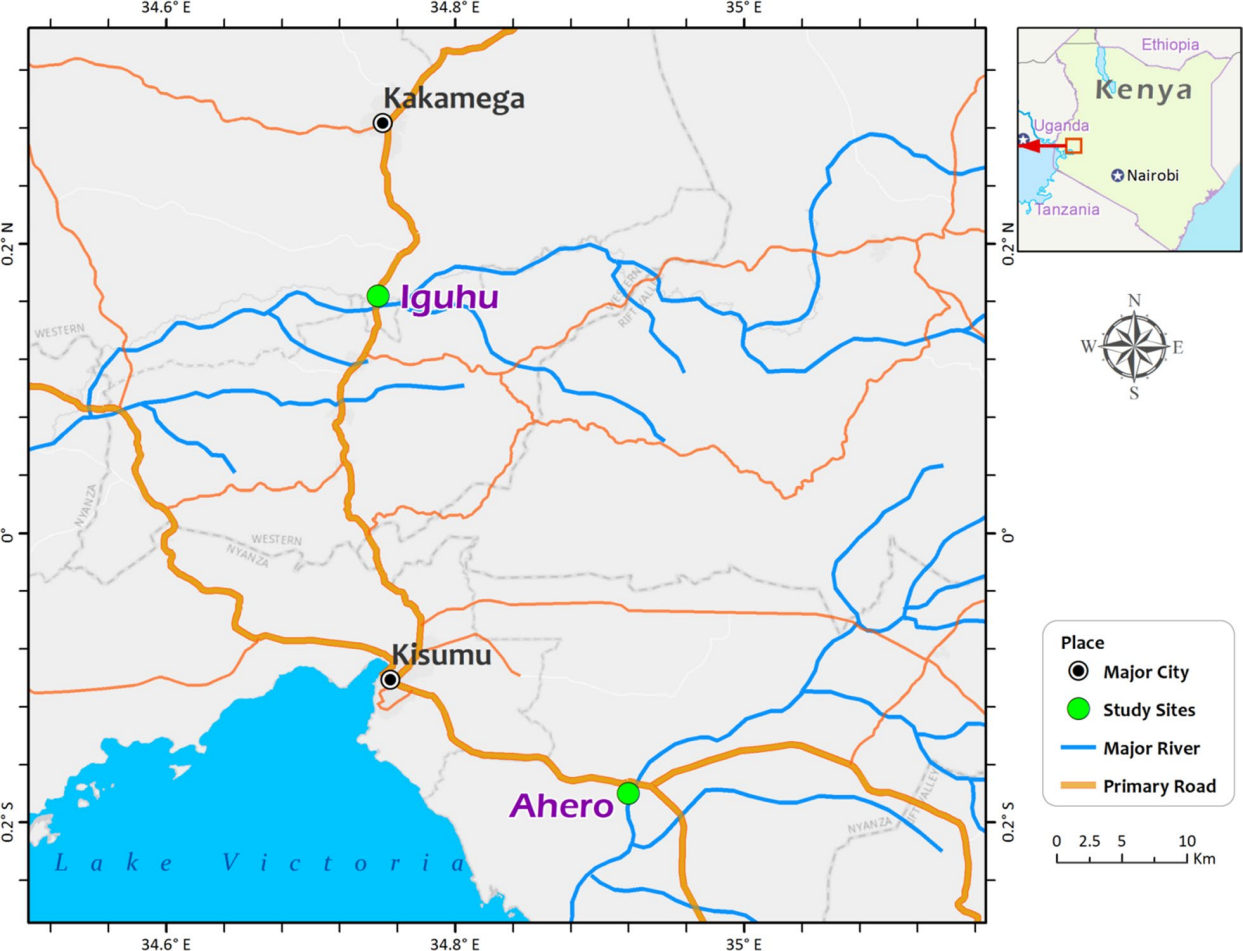
Map of the study sites

The study was conducted in lowland and highland settings of western Kenya. Two sites were selected (Fig. 1): Ahero (0°.11′S, 34°.55′E, altitude 1162 m) in Kisumu county and Iguhu (0°.17′N; 34°.74′E, altitude 1430–1580 m a.s.l) in Kakamega county. Iguhu site is highland characterized by valleys and depressions surrounded by densely populated hills whereas Ahero is lowland plain area. The sites have bimodal pattern of rainfall, with long rainy season from April to June, which triggers peak malaria transmission period and short rainy season from October to November with minimal transmission [24]. The hot and dry season is from January to March and this marks the lowest transmission [5]. *Plasmodium falciparum* is the predominant malaria species in the area and is transmitted by *An. gambiae* s.s., *An. arabiensis* and *An. funestus* [5, 25].

### Mosquito collections

Adult mosquito collections were carried out monthly during the short rainy season (September to November) in 2015 and dry season (February to April) in 2016. Indoor and outdoor host-seeking mosquitoes were collected using Centers for Disease Control and Prevention (CDC) light traps (John W. Hock Ltd, Gainesville, FL., USA). For indoor host-seeking mosquito collection, CDC light traps were set inside houses near the bed at a height of 1.5 m from 18:00 to 06:00 h in twenty randomly selected houses per month in each study site. For the outdoor host-seeking mosquito sampling, CDC light traps were set outdoor in the vicinity (within 2 m) of sentinel houses. The same houses were used for mosquito collections each month.

Indoor resting mosquitoes were sampled using pyrethrum spray catches (PSCs) from another twenty randomly selected houses from 06:00 to 09:00 h following standard protocol [26]. Outdoor resting mosquitoes were collected monthly in the mornings (06:00 to 09:00 h.) from twenty artificial outdoor pit shelters constructed according to the method of Muirhead-Thomson [27], in the compound of 20 selected houses in each study site. The collections were repeated using the same pit shelters each month.

Along with mosquito collection, data on the numbers of potential hosts in the study area including human, bovine, goat, dog and chicken were collected using questionnaire surveys. All collected mosquitoes were identified morphologically to species using keys [28]. Female *Anopheles* mosquitoes were further classified as unfed, blood fed, half-gravid and gravid. Each mosquito was kept in a labelled 1.5 ml Eppendorf tube containing silica gel desiccant and cotton wool. Samples were stored at − 20 °C refrigerator at Climate and Human Health Research Laboratory of Kenya Medical Research Institute until used for further processing.

### Identification of vector species complexes

Members of *An. gambiae* s.l. and *An. funestus* s.l. groups were identified to species by polymerase chain reaction (PCR), following the protocols developed by Scott et al. for *An. gambiae* s.l. [29] and Koekemoer et al. for *An. funestus* s.l. [30].

### Detection of blood meal sources

The blood meal sources of freshly fed *Anopheles* mosquitoes were analyzed by a direct enzyme-linked immunosorbent assay (ELISA) [31] using human, bovine, goat, chicken and dog antibodies. Positive controls were included for each host during the assay. Laboratory reared unfed *An. gambiae* was used as negative control.

### Sporozoite ELISA

Dried head and thorax of the preserved *Anopheles* mosquito specimens were carefully separated from the abdomen and tested for *P. falciparum* circumsporozoite proteins (CSPs) using sand-witch ELISA method [32, 33].

### Data analysis

The density of adult anopheline mosquitoes was calculated as the number of female mosquitoes per trap/night for each collection method. Analysis of variance (ANOVA) was used to compare malaria vector density between indoor and outdoor locations. χ^2^-test was employed to test the difference in vector species composition between indoor and outdoor.

Human blood index (HBI) was calculated as the proportion of *Anopheles* mosquitoes that fed on human over the total *Anopheles* tested for blood meal origins [34]. Bovine, goat, dog and chicken blood indices were also calculated in similar way. Mixed blood meals were included in the calculation of blood meal indices [35]. The forage ratio (FR), a measure of host preference by mosquitoes, was determined as the percent of engorged *Anopheles* mosquitoes which have fed on a given host (human, bovine, goat, dog or chicken) divided by the percent which it comprises in the total population of hosts available in the study area [36]. The FR *w*_*i*_ for species *i* was calculated as:

$$w_{i} = \frac{{o_{i} }}{{p_{i} }}$$

where *w*_*i*_ is the FR for mosquito species *i*, *o*_*i*_ is the proportion of host species *i* in the blood meals, and *p*_*i*_ is the proportion of host species *i* available in the environment.

Statistical significance of the FR estimate for each host was based on overlap of the 95% confidence interval (CI) of the estimate with the value one [37]. A host was considered to have been preferred if the lower 95% confidence limit for the FR estimate was greater than one. A host was inferred to have been avoided if the upper 95% confidence limit for the FR estimate was less than one. A host for which the 95% CI for its FR included one was considered to have been feed on opportunistically [37].

The sporozoite rate was estimated as the proportion of mosquitoes positive for *P. falciparum* CSPs over the total number tested. Annual entomological inoculation rate (EIR) was calculated from mosquito collections by CDC light traps using the formula, 1.605 × (no. CSP-positive ELISA results from CDC light traps/no. mosquitoes tested) × (no. mosquitoes collected from CDC light traps/no. trap-nights) × 365 [38, 39]. The multiplication factor 1.605 is a conversion factor for CDC light trap catches vs. man biting catches [38]. The annual EIR of *Anopheles* mosquitoes collected by PSCs was determined as: (no. fed mosquitoes caught by PSC/no. human occupants who spent the night in the sprayed house) × (no. mosquitoes fed on human/no. mosquitoes tested for human blood meal) × (PSC based sporozoite rate) × 365 [40].

The annual EIR for *Anopheles* mosquitoes collected from pit shelters was also estimated as (no. fed mosquitoes caught in the pit shelters/no. human occupants who spent the night in a house nearest to the pit shelter) × (no. human fed mosquitoes/no. mosquitoes tested for human blood meal) × (sporozoite rate from pit shelters) × 365. This formula was employed based on the assumption that all *Anopheles* mosquitoes collected from pit shelters have got their human blood meals from occupants of the nearest house, either indoor or outdoor.

Data were analyzed using STATISTICA 8.0 (StatSoft, Tulsa, USA) and SPSS version 20.0 (SPSS, Chicago, IL, USA) software packages. p < 0.05 was considered statistically significant during the analysis.

## Results

### Anopheline mosquito species composition and abundance

A total of 10,864 female *Anopheles* mosquitoes belonging to four species were collected during the study period (Table 1). *Anopheles gambiae* s.l. was the predominant species accounting for 71.4% of the total captures, followed by *An. funestus* s.l. (12.3%), *Anopheles coustani* complex (9.2%) and *Anopheles pharoensis* (7.1%). In addition, 3263 male anopheline mosquitoes and 5206 *Culex* species (males and females together) were collected over the study period. There was a significant difference in anopheline mosquito species co-occurrence between the study sites (*F*_1, 952_ = 423.02, p < 0.0001). There was also significant difference in anopheline mosquito species co-occurrence between indoor and outdoor locations (*F*_1, 956_ = 29.44, p < 0.0001). The majority (61.8%) of the anopheline mosquitoes were collected outdoors.

**Table 1:** Summary of female *Anopheles* mosquitoes collected from indoor and outdoor in lowland (Ahero) and highland (Iguhu) settings of western Kenya (n = 120 trap-nights for each trap)

Study sites and *Anopheles* spp.	Indoor		Outdoor		Total
	Light trap	PSC	Light trap	Pit shelter	
Ahero					
*An. gambiae* s.l.	1592	1009	1636	3262	7499
*An. funestus* s.l.	628	204	270	142	1244
*An. coustani*	321	2	652	15	990
*An. pharoensis*	78	0	688	0	766
Iguhu					
*An. gambiae* s.l.	108	51	56	41	256
*An. funestus* s.l.	49	30	13	4	96
*An. coustani*	3	0	10	0	13
Total	2779	1296	3325	3464	10,864

PSC, pyrethrum spray catch

### Indoor and outdoor *Anopheles* mosquito density

**Fig. 2:**
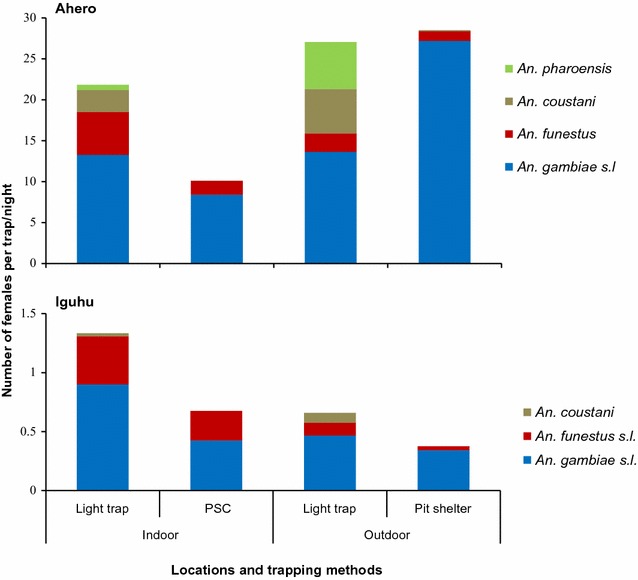
Indoor and outdoor host-seeking and resting density of female *Anopheles* mosquitoes collected from Ahero and Iguhu, western Kenya

Figure 2 shows the mean indoor and outdoor density of host-seeking and resting female *Anopheles* mosquitoes. In Ahero, the mean outdoor resting density of *An. gambiae* s.l. was significantly higher than indoor resting density (*t*_238_ = 8.45, p < 0.0001), whereas the difference in mean indoor and outdoor resting density of *An. funestus* s.l. was not significant (p > 0.05). The mean outdoor host-seeking density of *An. gambiae* s.l. was also higher than indoor, although the difference was not statistically significant (*t*_238_ = 0.14, p = 0.889). The mean indoor host-seeking density of *An. funestus* s.l. was significantly higher than outdoor (*t*_238_ = 2.37, p = 0.019). Significantly higher outdoor host-seeking density than indoor was observed for *An. coustani* (*t*_238_ = 2.589, p = 0.01) and *An. pharoensis* (*t*_238_ = 4.923, p < 0.0001).

In Iguhu, the indoor host-seeking densities of *An. gambiae* s.l. and *An. funestus* s.l. were significantly higher than outdoor (*An. gambiae* s.l., *t*_238_ = 2.12, p = 0.034; *An. funestus* s.l., *t*_238_ = 3.09, p = 0.002). The difference in mean indoor and outdoor resting density of *An. gambiae* s.l. was not significant (*t*_238_ = 0.97, p = 0.335), while the mean indoor resting density of *An. funestus* s.l. was significantly higher (*t*_238_ = 3.23, p = 0.001) than outdoor.

### Composition of *Anopheles gambiae* and *Anopheles funestus* sibling species

**Fig. 3:**
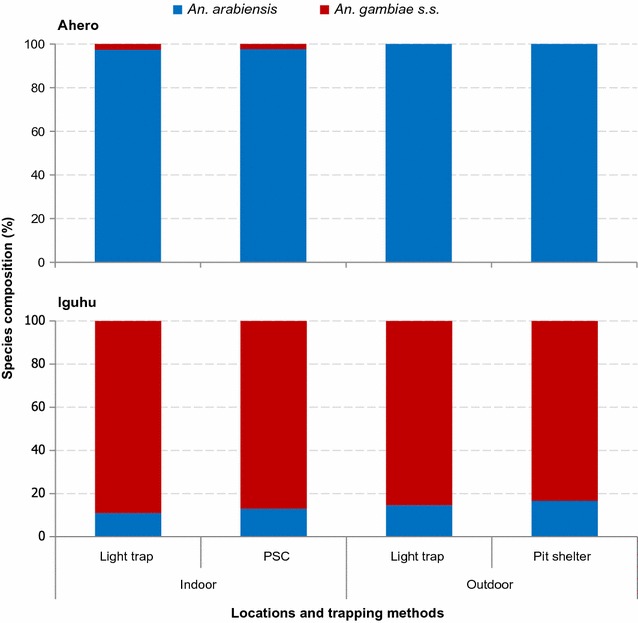
Composition of *Anopheles gambiae* sibling species in Ahero and Iguhu, western Kenya

A total of 750 specimens (628 *An. gambiae* s.l. and 122 *An. funestus* s.l.) were analysed for identification of their respective sibling species. Of theses, 581 *An. gambiae* s.l. and 108 *An. funestus* s.l. specimens were successfully amplified and identified to species by PCR. Figure 3 shows member species of *An. gambiae* s.l. In Ahero, of the *An. gambiae* s.l. assayed, *An. arabiensis* and *An. gambiae* s.s accounted for 98.9 and 1.1%, respectively. In contrast in Iguhu, *An. gambiae* s.s. and *An. arabiensis* constituted 87 and 13%, respectively of the assayed *An. gambiae* s.l. specimens. Overall, there was significant difference between indoor and outdoor locations in terms of *An. gambiae* s.l. species composition (χ^2^ = 26.443, *df* = 1, p < 0.0001). The proportion of *An. arabiensis* was higher outdoors than indoors. Of the 108 *An. funestus* s.l. confirmed by PCR, *An. funestus* s.s. (hereafter *An. funestus*) and *Anopheles leesoni*, accounted for 98.1 and 1.9%, respectively. All the *An. leesoni* were from outdoor CDC light traps. The member species of *An. funestus* s.l. did not vary between the study sites.

### Physiological status

In both indoor and outdoor collections, the majority (> 70%) of the host-seeking anophelines were unfed. About 55% of the indoor resting and 39% of the outdoor resting *An. arabiensis* were blood fed. One-third of the indoor resting and 31.7% of the outdoor resting *An. gambiae* s.s. were blood fed. About half of the indoor resting *An. funestus* were blood fed, while this was 11.6% for the outdoor resting *An. funestus.*

### Blood meal indices

Table 2 shows the host blood indices of *An. arabiensis* and *An. funestus* in Ahero. The HBI of *An. arabiensis* from indoor CDC light traps and PSCs was 8.2 and 1.2%, respectively, whereas the HBI of *An. arabiensis* from outdoor CDC traps and pit shelters was 3.4 and 0.7%, respectively. The overall HBI of *An. arabiensis* was 2.5%. The HBI of *An. funestus* from indoor CDC light traps and PSCs was 72.7 and 63.6%, respectively, while the HBI of *An. funestus* from both outdoor CDC light traps and pit shelters was 50%. In Ahero, the overall HBI for *An. funestus* was 62%.

**Table 2:** Blood meal origins of *An. arabiensis* and *An. funestus* from indoor and outdoor collections in Ahero, western Kenya

Blood-meal origins	*An. arabiensis*				*An. funestus*			
	Indoor		Outdoor		Indoor		Outdoor	
	Light trap	PSC	Light trap	Pit shelter	Light trap	PSC	Light trap	Pit shelter
Number tested	122	165	59	298	11	44	4	12
Human	7 (5.7)	1 (0.6)	2 (3.4)	2 (0.7)	8 (72.7)	23 (52.3)	2 (50.0)	6 (50.0)
Bovine	74 (60.7)	108 (65.5)	30 (50.8)	251 (84.2)	0	10 (22.7)	1 (25.0)	5 (41.7)
Goat	5 (4.1)	5 (3.0)	1 (1.7)	4 (1.3)	0	0	0	0
Dog	1 (0.8)	5 (3.0)	1 (1.7)	5 (1.7)	0	0	0	0
Chicken	2 (1.6)	0	0	1 (0.3)	0	0	0	0
Human + bovine	1 (0.8)	1 (0.6)	0	0	0	2 (4.6)	0	0
Human + dog	2 (1.6)	0	0	0	0	3 (6.8)	0	0
Bovine + dog	1 (0.8)	1 (0.6)	0	4 (1.3)	0	0	0	0
Goat + dog	0	1 (0.6)	0	0	0	0	0	0
Dog + chicken	0	0	0	1 (0.3)	0	0	0	0
Unknown	29 (23.8)	43 (26.1)	25 (42.4)	30 (10.1)	3 (27.3)	6 (13.6)	1 (25.0)	1 (8.3)
HBI	8.2	1.2	3.4	0.7	72.7	63.6	50.0	50.0

HBI was calculated as the number of mosquito positive for human (including mixed blood meal) divided by the total number tested

HBI, human blood index; PSC, pyrethrum spray catches

In contrast, the bovine blood index (BBI) of *An. arabiensis* from indoor CDC light traps, PSCs, outdoor CDC light traps and pit shelters was 62.3, 66.7, 50.8, and 85.6%, respectively. Overall, the BBI of *An. arabiensis* was 73.1%. The BBI of *An. funestus* from PSCs, outdoor CDC light traps and pit shelters was 27.3, 22.7 and 41.7%, respectively. None of the *An. funestus* from indoor CDC light traps was positive for bovine blood meal. In Ahero, the overall BBI of *An. funestus* was 25.4%. Blood meal indices for other vertebrate hosts (goat, dog and chicken) were low (< 4%).

Table 3 shows the host blood indices of *An. gambiae* s.s. and *An. funestus* in Iguhu. The HBI of *An. gambiae* s.s. from indoor CDC light traps and PSCs was 70.0 and 76.5%, respectively, whereas the HBI of *An. gambiae* s.s. from outdoor CDC light traps and pit shelters was 20.0 and 23.1% respectively. The overall HBI of *An. gambiae* s.s. was 50.0%. The HBI of *An. funestus* from indoor CDC light traps and PSCs was 53.8 and 61.1%, respectively. In outdoor CDC light traps, very small number of fed *An. funestus* was caught, which yielded a HBI of 50%. Hence, in Iguhu, the overall HBI of *An. funestus* was 55.9%.

**Table 3:** Blood meal origins of *An. gambiae* s.s. and *An. funestus* from indoor and outdoor collections in Iguhu, western Kenya

Blood-meal origins	*An. gambiae* s.s.				*An. funestus*			
	Indoor		Outdoor		Indoor		Outdoor	
	Light trap	PSC	Light trap	Pit shelter	Light trap	PSC	Light trap	Pit shelter
Number tested	10	17	10	13	13	18	2	1
Human	7 (70)	11 (64.7)	2 (20)	3 (23.1)	7 (53.8)	11 (61.1)	1 (50.0)	0
Bovine	0	3 (17.6)	4 (40)	6 (46.1)	2 (15.4)	3 (16.7)	1 (50.0)	0
Goat	0	0	0	0	1 (7.7)	0	0	0
Dog	0	0	0	1 (7.7)	1 (7.7)	0	0	1 (100)
Human + bovine	0	1 (5.9)	0	0	0	0	0	0
Human + dog	0	1 (5.9)	0	0	0	0	0	0
Unknown	3 (30)	1 (5.9)	4 (40)	3 (23.1)	2 (15.4)	4 (22.2)	0	0
HBI	70.0	76.5	20	23.1	53.8	61.1	50.0	0

HBI was calculated as the number of mosquito positive for human (including mixed blood meal) divided by the total number tested

HBI, human blood index; PSC, pyrethrum spray catches

The BBI of *An. gambiae* s.s. from PSCs, outdoor CDC light traps and pit shelters was 23.5, 40.0, and 46.1%, respectively. None of the tested *An. gambiae* s.s. from indoor CDC light traps was positive for bovine blood meal. The overall BBI of *An. gambiae* s.s. was 28%. The BBI of *An. funestus* from indoor CDC light traps, PSCs and outdoor CDC light traps was 15.4, 16.7, and 50%, respectively. In Iguhu, the overall BBI of *An. funestus* was 17.6%.

### Feeding preference of malaria vectors

The overall blood meal indices and host preferences of *Anopheles* mosquitoes are shown in Table 4. Regardless of higher proportion of humans compared to domestic animals in Ahero, *An. arabiensis* showed a strong preference to feed on bovine (Forage ratio, FR = 3.9, 95% CI 3.7–4.9). *Anopheles gambiae* s.s. showed preference to both human (FR = 1.8, 95% CI 1.3–2.3) and bovine (FR = 2.3, 95% CI 1.3–3.3). *Anopheles funestus* showed a preference to human in both Ahero (FR = 2.2, 95% CI 1.8–2.6) and Iguhu (FR = 2.0, 95% CI 1.6–2.4).

**Table 4:** Overall blood meal indices and host-preferences of malaria vectors from indoor and outdoor collections in Ahero and Iguhu, western Kenya

Site and species	Parameters	Human	Bovine	Goat	Dog	Chicken
Ahero						
Host abundance in the area (%)		27.8	18.8	4.0	6.0	43.4
*An. arabensis*	Blood index	2.5	73.1	2.5	3.4	0.6
	FR (95% CI)	0.09 (0.05 to 0.13)	3.9 (3.7 to 4.1)*	0.6 (0.3 to 0.9)	0.5 (0.3 to 0.7)	0.01 (− 0.03 to 0.05)
*An. funestus*	Blood index	62.0	25.4	0	4.2	0
	FR (95% CI)	2.2 (1.8 to 2.6)*	1.4 (0.9 to 1.9)	0	0.7 (− 0.1 to 1.5)	0
Iguhu						
Host abundance in the area (%)		27.5	12.4	2.4	2.5	55.2
*An. gambiae* s.s.	Blood index	50	28	0	2.0	0
	FR (95% CI)	1.8 (1.3 to 2.3)*	2.3 (1.3 to 2.3)*	0	0.8 (− 0.7 to 2.3)	0
*An. funestus*	Blood index	55.9	17.6	2.9	2.9	0
	FR (95% CI)	2.0 (1.6 to 2.4)*	1.4 (0.4 to 2.4)	1.2 (− 1.2 to 3.6)	1.2 (− 1.1 to 3.5)	0

FR, forage ratio

* Indicates the preferred host

### Sporozoite rates

Overall, 2608 *Anopheles* mosquitoes comprising *An. arabiensis* (n = 1280), *An. gambiae* s.s. (n = 214), *An. funestus* (n = 629), *An. coustani* (n = 255) and *An. pharoensis* (n = 230) were tested for *P. falciparum* CSPs. Of these, 20 specimens (2 *An. arabiensis*, 5 *An. gambiae* s.s., 12 *An. funestus* and 1 *An. coustani*) were positive for CSPs.

Table 5 shows the sporozoite rates of *Anopheles* mosquitoes collected from indoors and outdoors. In Ahero, the sporozoite rate of *An. arabiensis* from indoor and outdoor CDC light traps was 0.38 and 0.35%, respectively. However, none of the *An. arabiensis* tested from PSCs and pit shelters were positive. The overall sporozoite rate of *An. arabiensis* was 0.16%. The sporozoite rate of *An. funestus* from indoor CDC light traps and PSCs was 2.6 and 2.0%, respectively, while this was 1.2% from both outdoor CDC light traps and pit shelters. Hence, in Ahero, the overall spozoite rate of *An. funestus* was 1.8%. Moreover, one *An. coustani* specimen from outdoor CDC light trap was positive for CSP.

**Table 5:** Sporozoite rates of *Anopheles* mosquitoes from indoor and outdoor collections in Ahero and Iguhu, western Kenya

Study site and *Anopheles* sp.	Parameters	Indoor		Outdoor		Total
		Light trap	PSC	Light trap	Pit shelter	
Ahero						
*An. arabiensis*	No tested	263	264	286	447	1260
	Pf +ve (%)	1 (0.38)	0	1 (0.35)	0	2 (0.16)
*An. funestus*	No tested	194	100	169	84	547
	Pf +ve (%)	5 (2.6)	2 (2.0)	2 (1.2)	1 (1.2)	10 (1.8)
*An. coustani*	No tested	50	0	200	0	250
	Pf +ve (%)	0	0	1 (0.5)	0	1 (0.4)
*An. pharoensis*	No tested	25	0	205	0	230
	Pf +ve (%)	0	0	0	0	0
Iguhu						
*An. gambiae* s.s.	No tested	84	46	50	34	214
	Pf +ve (%)	3 (3.6)	0	1 (2.0)	1 (2.9)	5 (2.3)
*An. funestus*	No tested	42	25	13	2	82
	Pf +ve (%)	1 (2.4)	1 (4.0)	0	0	2 (2.4)
*An. arabiensis*	No tested	8	5	2	5	20
	Pf +ve (%)	0	0	0	0	0
*An. coustani*	No tested	1	0	4	0	5
	Pf +ve (%)	0	0	0	0	0

*Pf*, *Plasmodium falciparum*; *Pf* +ve, number *P. falciparum* CSP positive (rate in percent)

In Iguhu, the sporozoite rate of *An. gambiae* s.s. from indoor CDC light traps was 3.6%, but none of the *An. gambiae* s.s. tested from PSCs was positive. In contrast, the sporozoite rate of *An. gambiae* s.s. from outdoor CDC light traps and pit shelters was 2.0 and 2.9%, respectively. Overall, the sporozoite rate of *An. gambiae* s.s. was 2.3%. The sporozoite rate of *An. funestus* from indoor CDC light traps and PSCs was 2.4 and 4%, respectively. No CSP was detected in *An. funestus* collected from outdoor CDC light traps and pit shelters. Thus, in Iguhu, the overall sporozoite rate of *An. funestus* was 2.4%.

### Entomological inoculation rates (EIRs)

The EIRs of *Anopheles* mosquitoes are shown in Table 6. In Ahero, the estimated *P. falciparum* EIR of *An. arabiensis* from indoor and outdoor CDC light traps was 29.6 and 27.9 infective bites/person/year (ib/p/year), respectively, whereas the EIR of *An. funestus* from indoor and outdoor CDC light traps was 79.0 and 15.6 ib/p/year, respectively. The overall indoor and outdoor EIR was 108.6 and 43.5 ib/p/year, respectively. About 48% of the total infective bites by *An. arabiensis* and 16.5% by *An. funestus* occurred outdoor. The EIR of *An. arabiensis* and *An. funestus* from PSCs was 0 and 0.92 ib/p/year, respectively.

**Table 6:** Entomological inoculation rates (EIR) of malaria vectors from indoor and outdoor collections in Ahero and Iguhu, western Kenya

Site and species	Parameters	Indoor		Outdoor	
		Light trap	PSC	Light trap	Pit shelter
Ahero					
*An. arabiensis*	SR	0.38	0	0.35	0
	EIR	29.6	0	27.9	0
*An. funestus*	SR	2.6	2.0	1.2	1.2
	EIR	79.0	0.92	15.6	0.05
*An. coustani*	SR	0	0	0.5	0
	EIR	0	0	16.8	0
Iguhu					
*An. gambiae* s.s.	SR	3.6	0	2.0	2.9
	EIR	18.8	0	5.5	0.17
*An. funestus*	SR	2.4	4.0	0	0
	EIR	5.7	0.82	0	0
*An. arabiensis*	SR	0	0	0	0
	EIR	0	0	0	0

SR, sporozoite rate in percent; EIR, annual entomological inoculation rate measured as the number of infective bites/person/year; PSC, pyrethrum spray catch

In Iguhu, the estimated *P. falciparum* EIR of *An. gambiae* s.s. from indoor and outdoor CDC light traps was 18.8 and 5.5 ib/p/year, respectively, whereas the EIR of *An. funestus* from indoor and outdoor CDC light traps was 5.7 and 0 ib/p/year, respectively. The overall indoor and outdoor EIR was 24.5 and 5.5 ib/p/year, respectively. About 22.6% of the total infective bites by *An. gambiae* s.s. occurred outdoor. The EIR of *An. gambiae* s.s. and *An. funestus* from PSCs was 0 and 0.82 ib/p/year, respectively.

## Discussion

This study showed that *An. arabiensis* was the most abundant anopheline species in Ahero (lowland), whereas *An. gambiae* s.s. was the most abundant species in Iguhu (highland) sites of western Kenya. *An. funestus* was the second most abundant species in both sites, which is consistent with previous studies [5, 6].

*Anopheles arabiensis* showed increased exophagic tendency in the study area when compared with the findings of studies conducted before the scale up of vector control interventions [22, 41]. For instance, studies by Githeko et al. in 1990s, when ITN coverage was negligeable, showed that *An. arabiensis* was two times more likely to bite indoors than outdoors [22]. In the present study, the outdoor biting density of *An. arabiensis* was higher than indoor. The increased outdoor host-seeking tendency of *An. arabiensis* in this study compared to the previous reports might be due to the scale-up of ITNs. Bayoh et al. also noted that *An. arabiensis* was more likely to bite outdoors in western Kenya when compared with data collected before the scale-up of ITNs [19]. Moreover, *An. arabiensis* showed highly exophilic behaviour in this study, with significantly higher outdoor resting density than indoor resting density.

The proportion of *An. arabiensis* has been increasing in western Kenya highlands. Until 2002, *An. gambiae* s.s. was the only member of *An. gambiae* s.l. complex reported in western Kenya highlands > 1400 m a.s.l. [25, 42]. The proportion of *An. arabiensis* was reported to be 0.8% in 2003 [43] and reached 9.2% in 2010 [5]. In this study, the proportion of adult *An. arabiensis* increased to 13%. A recent study reported a higher proportion of *An. arabiensis* (38.2%) in larval population [44]. The continued proportional increase in *An. arabiensis* population might be due to the increased ITN coverage [9, 10] and/or the zoophilic and exophagic/exophilic behaviour of this species or due to species shift. Other factors such as climatic and environmental change, which resulted in increased temperature or availability of more habitats in the area, might have also contributed as this was found to favor *An. arabiensis* [45]. Such shift in vector species composition could undermine the efficacy of ITNs as the interventions do not target zoophilic and exophilic vector species which avoids the lethal effect of ITNs and sustain residual malaria transmission [46].

*Anopheles gambiae* s.s. showed endophagic behaviour, with higher indoor host-seeking density than outdoor. This is in agreement with the earlier reports by Githeko et al. [22]. Recent studies in western Kenya have also showed that *An. gambiae* s.s. was more likely to seek hosts indoor than outdoor [19, 47]. In contrast, studies in Bioko Island, Equatorial Guinea showed that *An. gambiae* s.s. seek hosts outdoor than indoor [48]. This difference might be due to the variation in molecular forms of *An. gambiae* s.s. (S and M/*Anopheles coluzzii*) from Kenya and Equatorial Guinea [49] although the variability in host-seeking behaviour between the two molecular forms is not yet explicitly described.

It is unusual that *An. gambiae* s.s. showed similar feeding preference to human and bovine. Two decades ago, the HBI of indoor resting *An. gambiae* s.s. in western Kenya and other parts of the country was 96–97%, an indication that they had fed exclusively on humans [21, 23, 50]. In this study, the overall HBI of *An. gambiae* s.s. was only 50.0% although predominantly from indoor collection. Compared to the earlier studies conducted in western Kenya before ITNs were used in large scale [21, 23], the HBI of indoor resting *An. gambiae* s.s. has significantly dropped by 20% and the drop was entirely replaced by BBI. For outdoor resting *An. gambiae* s.s., the BBI reached up to 46%. Similar reduction in HBI and increment in BBI has also been reported recently [7, 15]. This suggests an increasing tendency of *An. gambiae* s.s. to feed on bovine following the increased ITN coverage in the western Kenya highlands.

*Anopheles funestus* s.s. was the predominant species among *Anopheles funestus* group in the study area. Similar findings were reported in Tanzania [51]. Kweka et al. [52] also found that *An. funestus* s.s. was the predominant sibling species in larvae population in western Kenya. However, there was significant difference in terms of the relative proportion of *An. funestus* s.s. between adult and larvae population. In this study, *An. funestus* s.s. accounted for 98.1% of the adult *An. funestus* s.l. population. In contrast, Kweka et al. found only 32.9% *An. funestus* s.s. in larvae population. This difference could be due to the presence of other zoophilic and exophilic sibling species of *An. funestus* s.l. in the larvae that do not bite or rest indoor or around human dwellings.

*Anopheles funestus* showed anthropophagic behaviour in both study sites, feeding predominantly on human. The anthrophagic behaviour of *An. funestus* was frequently observed in Kenya [21, 50] and elsewhere in Africa [53–56]. Nevertheless, they also fed on bovine, with higher BBI than the previous reports [21, 50, 53–56].

The secondary vectors, *An. pharoensis* and *An. coustani* showed exophagic behaviour, with significantly higher outdoor host-seeking density than indoor. Other studies in Kenya [41, 57] and elsewhere in Africa [58–60] reported similar phenomenon for these species. It is worth mentioning that both *An. pharoensis* and *An. coustani* were very rare in both indoor resting collections and pit shelters despite their preponderance in CDC light traps. Hence, further studies are required to find out the potential resting places of *An. pharoensis* and *An. coustani.*

The EIR data showed that the majority of malaria transmission by *An. gambiae* s.s. and *An. funestus* occurred indoors, while *An. arabiensis* contributed almost equally to both outdoor and indoor transmission. The higher indoor EIRs despite high ITN coverage could be attributed to inconsistent ITN use [61], increasing insecticide resistance among vectors [5, 8], and shifts in malaria vector biting times from mid-night to early evening and morning when people are still indoor but unprotected by ITNs [47, 62]. However, the magnitude of the outdoor EIRs was also considerably high compared to previous reports [19]. The ongoing shifts in vector species composition and changes in vector behaviours might have contributed to the high outdoor EIRs.

In addition to the primary vectors, a single specimen of *An. coustani* from outdoor CDC light trap was found to be positive for *P. falciparum* CSP based on ELISA, although not yet confirmed by PCR. Studies are increasingly reporting the importance of the secondary vectors in residual malaria transmission [57, 60, 63–65]. Several studies have demonstrated the susceptibility of *An. coustani* to *P. falciparum* infection [57, 59, 60, 66]. Although ELISA technique is not specific enough to incriminate zoophagic mosquitoes as a vector [33], a recent study in Madagascar confirmed the presence of *Plasmodium* CSP in *An. coustani* by both ELISA and PCR [60], suggesting that this species could play a role in outdoor malaria transmission.

## Conclusion

*Anopheles arabiensis* was highly exophilic and zoophagic*. Anopheles gambiae* s.s. showed an increasing tendency to feed on bovine while *An. funestus* showed anthropophagic behaviour. While most of malaria transmission occurred indoor, the magnitude of outdoor transmission was considerably high. Additional control tools that complement the existing interventions are required to control residual transmission. Further studies are required to comprehend the role of secondary vectors in malaria transmission.

## Abbreviations

BBI: bovine blood index
CDC: Centers for Disease Control and Prevention
CSP: circumsporozoite protein
EIR: entomological inoculation rate
ELISA: enzyme-linked immunosorbent assay
FR: forage ratio
HBI: human blood index
IRS: indoor residual spray
ITN: insecticide-treated net
PCR: polymerase chain reaction
PSC: pyrethrum spray catch
